# Source Case Investigation for Children with TB Disease in Pune, India

**DOI:** 10.1155/2014/182836

**Published:** 2014-08-27

**Authors:** Debalina De, Aarti Kinikar, P. S. Adhav, Sunanda Kamble, Prasanna Sahoo, Hari Koli, Savita Kanade, Vidya Mave, Nishi Suryavanshi, Nikhil Gupte, Amita Gupta, Jyoti Mathad

**Affiliations:** ^1^University of Michigan Medical School, 1301 Catherine Road, Ann Arbor, MI 48109, USA; ^2^Byramjee Jeejeebhoy Medical College/Sassoon General Hospital, Jai Prakash Narayan Road, Pune, Maharashtra 411001, India; ^3^Byramjee Jeejeebhoy Medical College/Johns Hopkins Clinical Trials Unit, Jai Prakash Narayan Road, Pune, Maharashtra 411001, India; ^4^Division of Infectious Diseases, Johns Hopkins University School of Medicine, 600 North Wolfe Street, Baltimore, MD 21287, USA; ^5^Division of Infectious Diseases, Weill Cornell Medical College, 525 E 68th Street, New York, NY 10021, USA

## Abstract

*Setting.* Contact tracing is broadly encouraged for tuberculosis (TB) control. In many high-burden countries, however, little effort is made to identify contacts of newly diagnosed TB patients. This failure puts children, many of whom live in poor crowded communities, at special risk. *Objectives.* To perform source-case investigations for 50 pediatric TB cases in Pune, India. *Design.* A descriptive cross-sectional observational study of pediatric TB cases < 5 years of age. Information was collected about the index case and household contacts. *Results.* In 15 (30%) of the 50 pediatric index cases, the household contained known TB contacts, 14 (86%) of whom were adults. Prior to their own diagnosis of TB, only one of the 15 pediatric index cases who met criteria for isoniazid preventive therapy received it. The index cases with known household TB contacts had a longer delay in initiating TB treatment than those without TB contacts (17.5 versus 2 days; *P* = 0.03). Use of contact tracing identified 14 additional household TB suspects, 8 (57%) of whom were children. *Conclusions.* This study identified missed opportunities for TB prevention, as contact tracing is poorly implemented in resource-limited countries, like India. Further strategies to improve the implementation of TB prevention, especially in young children, are urgently needed.

## 1. Introduction

Over 500,000 children were newly diagnosed with tuberculosis disease (TB) in 2012, and 74,000 died [1]. Diagnosing TB is especially challenging in children, because of their inability to produce adequate sputum samples and the paucibacillary nature of their disease—less than 15% of childhood TB is acid-fast smear positive and only 30–40% of cases are culture-confirmed [2]. This makes prevention crucial.

Contact with an active TB case may lead to TB disease, latent TB infection (LTBI), or TB exposure without infection. Those with newly acquired LTBI are at high risk of progression to disease within 2 years of infection without isoniazid preventive therapy (IPT), especially if they are children under the age of 5. The risk appears to decrease with increasing age: one-year-old children have a 50% risk of disease progression, 1-2-year-olds have a 20–25% risk, 2–5-year-olds have a 5% risk, and 5–10-year-olds have less than 2% risk [3]. The maximal benefit, then, is diagnosing and treating children 5 years of age or younger with primary TB infection.

The World Health Organization (WHO) and U.S. Centers for Disease Control and Prevention (CDC) recommend contact tracing to identify and offer treatment to people who have been in close contact with a confirmed TB source case [5, 6]. The recommendation is particularly important in pediatric cases, which are likely the result of new exposure rather than reactivation and, therefore, represent ongoing TB transmission in the community [2].

In low TB-burden countries like the U.S, all asymptomatic children living in the same home as someone with TB disease are offered IPT [6]. India, which accounts for 16% of global pediatric TB cases [1], has a similar policy, but the large migrant population, sparse diagnostic facilities, poor general knowledge of the disease, and limited public health infrastructure make contact tracing difficult [7]. IPT, therefore, is also offered inconsistently.

In this study, we performed source case investigations for children newly diagnosed with TB disease and assessed their household contacts for TB in a Western region of India to determine if the source was still infectious and if others in the household had been infected.

## 2. Study Population and Methods

### 2.1. Study Population

Between August 2010 and April 2011, we performed a descriptive observational cross-sectional study of children ≤5 years who were diagnosed with active TB. The study was conducted through Sassoon General Hospital, a 1300-bed government hospital in Pune, India, that cares for underserved populations in the surrounding urban and rural communities. We also enrolled participants from surrounding municipality clinics (Pune, Pimpri, and Chinchwad districts). In Pune district, the incidence of active TB in adults is 185 per 100,000 and the prevalence is 220 per 100,000. We included two groups in this study.

#### 2.1.1. Pediatric Index Cases

The inclusion criteria for this study were as follows: (1) age < 5 years (2) receipt of a regimen of first-line TB drugs within two months prior to enrollment, (3) meeting WHO criteria for confirmed, probable, or possible TB case (see below for definitions), (4) parents or legal guardians providing informed consent for the study participants, including consent for a household visit.

#### 2.1.2. Children and Adults in the Households of the Pediatric Index Cases

We obtained information regarding children and adults in the households of the pediatric index cases from the parent or legal guardian who provided consent. Information gathered on household members included TB diagnosis, method of TB diagnosis, TB treatment status, and HIV status.

The IRB and ethics committee at Byramjee Jeejeebhoy Medical College, Sassoon General Hospital, and Johns Hopkins University approved the study.

### 2.2. Methods

Trained social workers and research investigators visited the households of pediatric index TB cases. The research investigators administered questionnaires to the parent or legal guardian. Data regarding the demographics, current health (including HIV status, mode of TB diagnosis, and TB treatment), food security [8], and nutritional status for the pediatric index TB case were collected. Surveyors also inquired about the family income, the condition of the household (e.g., number of rooms, cook stove, and number of windows), and the health of other household members (e.g., presence of TB or symptoms of TB). HIV status was confirmed by the index case's medical record (e.g., DOTS card, ART card, or HIV test report). HIV status and results of TB tests (e.g., tuberculin skin test (TST), sputum test, and/or chest radiograph) were also recorded for the suspected household cases, if available.

#### 2.2.1. TB Definitions Used for Patients with at Least One Sign/Symptom of TB [9]

*(1) Confirmed TB Case*. A positive culture for* Mycobacterium tuberculosis* or a positive WHO-approved rapid diagnostic (WRD) test.

*(2) Probable TB Case*. Chest radiography consistent with TB and at least one of the following: a positive response to anti-TB treatment, documented exposure to someone with TB, or immunologic evidence of TB infection. Children with positive sputum or gastric aspirate specimen for acid-fast bacilli (AFB) are included in this category.

*(3) Possible TB Case*. Patient without confirmed TB who is receiving TB treatment, based on clinical diagnosis.

#### 2.2.2. TB Classification Definitions Used for Nonindex Household Cases (Defined by Study Investigators)

*(1) TB Source Case*. Any household member found to have TB disease (previously known and previously unknown cases) during a period when the child could have been exposed.

*(2) TB Suspects*. Household member with TB symptoms (cough, loss of appetite, night sweats, fever, and weight loss). These patients had not yet received a TB diagnosis and were not on any TB treatment.

### 2.3. Data Analysis

Data were entered into an Access database at Sassoon General Hospital and analyzed using Stata Version 12.0 (StataCorp, College Station, Texas). Simple statistical measures such as finding percentages, mean, median, and interquartile range were used to quantify the data. For comparison of categorical variables (e.g., urban residence and BCG vaccination status) between pediatric index cases with known TB cases versus those without and HIV-positive versus HIV-negative children, the chi-square or Fisher's exact test was used. For comparison of continuous variables (e.g., number of adults in the house), the medians were compared using Wilcoxon rank-sum test and means were compared using Student's *t*-test. All *P* values were two-sided with statistical significance evaluated at the 0.05 alpha level.

## 3. Results

During the study period, we performed household visits for 50 pediatric index cases. Of these cases, 20 were enrolled from Sassoon General Hospital and 30 from municipality DOTS centers in Pune/Pimpri/Chinchwad districts.

### 3.1. Study Population Characteristics

#### 3.1.1. Index Pediatric Case Characteristics

Fifty-eight percent (29/50) of the pediatric index cases in the study were boys. The median age of these cases was 27 months (IQR: 18–38). Seventy-two percent (36/50) of the cases reported being in close contact (close proximity with a person for more than one hour) with people outside of the household on a daily basis. The median time between TB diagnosis and the start of a regimen of first-line drugs was significantly higher in the group of pediatric index cases with known family TB contacts compared to those without any known family TB contacts (17.5 versus 2 days, *P* = 0.03) (Table 1).

At the time of TB diagnosis, 18% (9/50) of the pediatric index cases were HIV-positive, 72% (36/50) were HIV-negative, and 10% (5/50) did not know their HIV status (Table 2). Overall, 38% (19/50) of the cases had been diagnosed with pulmonary TB alone, 34% (17/50) with extrapulmonary TB alone, and 24% (12/50) with more than one type of TB (four percent (2/50) had no recorded type of TB). The most common form of extrapulmonary TB was abdominal, affecting 38% (10/26) of the pediatric index cases with extrapulmonary TB. Compared to HIV-negative cases, a higher percentage of HIV-positive children had multiple types of TB, though this did not reach statistical significance (Table 2). Ten percent (5/50) of the pediatric index cases had probable tuberculosis (4 sputum smear positive and 1 gastric aspirate smear positive); none of them were HIV-positive. The other most commonly used tests to diagnose TB included chest radiograph (54%, 27/50), tuberculin skin test (52%, 26/50), and abdominal ultrasound (44%, 22/50).

Ninety-six percent (48/50) of all pediatric index cases were currently receiving TB treatment at the time of the household visit. Fourteen percent (7/50) of the index cases had parents/guardians who indicated difficulty in bringing home the weekly course of TB medication; six of the seven were HIV-negative (Table 2). Of these seven, 57% (4/7) cited disruption of employment as the main barrier, 14% (1/7) reported far distance to DOTS center, 14% (1/7) reported cost of transportation to DOTS center, and 14% (1/7) reported confusion regarding retrieval of the TB medications (e.g., where to pick up the medications from and how often to go).

#### 3.1.2. Characteristics of Households and Household Contacts

The median number of people living in each household, including the index child, was similar in the pediatric index cases with known TB contacts and in those cases without any known TB contacts (Table 1). A total of 253 household members resided with the 50 pediatric index cases with 73% (185/253) being adults and 27% (68/253) being children between 0 and 18 years of age. The majority (76%) of the other children living in the home were less than 5 years old. Most households (92%, 46/50) were in urban/periurban slum dwellings with 84% (42/50) having two rooms or less and 64% (32/50) having no windows. Almost half of the households had at least one person employed for pay. The mean monthly household income was similar in household with and without known TB contacts—Rs. 6014 (~$124 USD) (IQR: Rs 3000–9000) and Rs. 6106 (~$126 USD) (IQR: Rs 3650–7500), respectively—about 50% lower than the mean household income in Pune [10]. However, 46% (7/15) of households with TB contacts had moderate to severe food insecurity versus only 14% (5/35) of homes without known TB contacts (*P* = 0.01) (Table 1).

#### 3.1.3. Characteristics of Prevalent Household TB Source Cases

Thirty percent (15/50) of the pediatric index cases enrolled in the study had at least one other previously known prevalent TB case in the household. One case had two confirmed household TB cases making the total number of confirmed known household TB source cases 16 (Table 3). Eighty-eight percent (14/16) of the confirmed household source cases were adults—37% (6/16) maternal caregivers, 31% (5/16) paternal caregivers, and 18% (3/16) other family members. Twelve percent (2/16) of the confirmed household source cases were other children, 6 and 7 years old, residing in the same household as the pediatric index case. Twenty-five percent (4/16) of the prevalent TB cases were HIV-positive adults. Ninety-three percent (15/16) of the confirmed household TB cases had initiated TB treatment. Only one out of the fifty pediatric index cases (2%) with a confirmed household contact reported receipt of IPT prior to his own diagnosis, though this child's guardian did not confirm adherence.

#### 3.1.4. Contact Tracing Results for New TB Suspects

Household contact tracing of the 50 pediatric index cases allowed for the identification of an additional 14 household members who met the definition of a suspected TB case (12 had cough for more than two weeks and two had fever). The 14 suspects came from the homes of 12 index cases, including two index cases with two suspected cases in each of their homes (Table 3). Fourteen (5.5%) of the total 253 individuals in the households met the criteria for a suspected TB case. Unlike the confirmed cases, who were mainly adults, more than half of the 14 TB suspected household cases were other children in the household (57% (8/14) children versus 43% (6/14) adults). Only 14% (2/14) were HIV-positive adults. The median age of the pediatric TB suspects was 4 years (IQR 2–6). None of the adult or pediatric TB suspects had previously received any form of TB treatment or prophylaxis. All TB suspects were recommended to receive further evaluation at the TB clinic at Sassoon Hospital. Of the combined 253 members residing in the same households as the enrolled participants, excluding the index children, 70% (177/253) had not been previously tested for tuberculosis (124 adults and 53 children).

## 4. Discussion

Failure to conduct adequate contact investigation represents a missed opportunity to prevent TB transmission to children. Living in the same household as someone with TB doubles a child's risk of death and increases it by eight times if the source case is the young child's mother [11]. Contact tracing allows for the identification of other TB suspects in the household so that they can be evaluated for or be given prophylaxis against active TB. Our source case study shows that contact investigation remains an important tool for TB control. Thirty percent of the children in our study had known source cases in their homes. Prior contact investigation may have prevented these cases of pediatric TB. We also identified 14 new suspected household TB cases (six adults and eight children), who were referred for further TB evaluation.

The poor implementation of IPT for children in our study is consistent with other data. In South India, only 19% of children <6 years identified through contact tracing of adult index cases were initiated on IPT. When treatment was offered, the adult was responsible for administering it to the child without follow-up for tolerance or adherence [12]. The situation is not unique to India. In Malawi, only 6% of children <5 years with a known household TB source case received IPT [13]. Similar practices have been documented in South Africa [14], Ethiopia [15], and Laos, where none of 148 children with household TB contacts received IPT [16]. Even in the United States, provision of IPT to children is incomplete, especially in immigrant families who have the highest risk of disease [17]. One study found that 75% of 2660 children and adolescents diagnosed with active TB in the United States had possible TB exposures prior to diagnosis but had never been screened or treated for LTBI [18].

While there is value to household contact tracing, protocols must be consistent with the lifestyle of the target population. In our population, for example, we were unable to identify a source in 70% (35/50) of the cases. The yield of source case investigations in the literature is variable. A study done in the United States found similar results to ours; only 37% of children with TB had source cases identified in their homes [17]. A study from New York City identified only 2 source cases out of 207 pediatric cases investigated [19]. Though the prevalence of TB is much lower in the United States, both American studies noted that including contacts outside the home would increase source case yields. In a study done in India, 38% of source cases for children with TB were from outside the family [20]. Similarly, in our study, many children residing in urban slum communities may have been exposed to TB outside the home. Indeed, 74% of families without known household TB in our study reported that the pediatric case was in daily contact with people outside of the immediate household, many at school. A study in Pakistan found that children who spent four to seven hours daily, the approximate length of a school day, with a TB case were more likely to acquire the disease [21]. Outbreaks of TB among school children in low-endemic countries have been well-described [22, 23]. A study in Italy reported traveling on the same bus as a pediatric index case increased the risk of a child developing TB infection over five-fold, higher than the risk accrued from living in the same area of residence as the index case [24].

Frequent, intense contact with people outside the family is even more pronounced in crowded urban slums worldwide. Many Indian children ride to school with 6 to 10 other children in an autorickshaw designed for two or three. Public buses in these settings also carry far more people than there are seats. Crowded environments outside the home may explain why, in South Africa, 45% with culture-confirmed TB had no household members with TB. Even among children with an active TB household contact, 36% had a strain that matched that of their community not of their household contact [25]. Contact tracing procedures must therefore account for duration, intimacy, and frequency of potential TB contacts beyond the household [26].

Contact investigation continues to play an important role in the prevention of TB transmission. Household visits provide an opportunity to educate members of the family and the community about TB and TB transmission. Community education efforts inform parents on how TB presents in children, decrease the stigma associated with TB, and encourage people to access healthcare earlier [27]. Furthermore, additional strategies to improve identification of possible contacts should be considered. For example, adding extra lines on DOTS cards for the names and ages of household members might assist in identifying children who have been exposed and may qualify for IPT. Physicians could also ask about potential TB contacts outside of the household (e.g., at work or school) to increase the yield of contact investigation. Modifications to the current protocol like these may be more efficacious in reducing TB transmission in TB-endemic countries, including India.

India's Revised National TB Control Program's (RNTCP) protocol states that asymptomatic children under 6 years of age who are from the same household as an adult with AFB smear- positive TB should be given six months of isoniazid (5 mg/kg daily) chemoprophylaxis [28], echoing the WHO recommendations [29]. That guideline, however, is unlikely to be followed without proactive contact tracing.

Our survey suggests that few children residing primarily in urban slums—the very places where TB is most common—receive appropriate TB preventive treatment when members of their household are diagnosed. Only one (7%) pediatric index case out of the 15 who were eligible for IPT had received IPT in accordance with RNTCP guidelines. To make matters worse, this child's guardian indicated difficulty in adherence with the present active TB treatment regimen. This demonstrates the role that poor TB knowledge and economic barriers play in in the TB epidemic in India. Despite the presence of a known TB contact in the household, children in these households had a significantly longer delay between diagnosis and treatment than those in households without TB contacts. The reason behind this is unclear. Because of the small size of the study, this could be related to selection bias. However, in a similar study in South Africa, 16% of children who were notified of having TB never began TB treatment. In that study, significantly more children who did not initiate TB treatment were from “squatter” communities, raising the possibility that unstable living conditions contribute to the low prioritization of medical issues [30]. Children often present with milder symptoms of TB masking the urgent need for treatment, especially for families facing financial hardships. Notably, in our study, households with prevalent TB cases also had a higher prevalence of food insecurity, another marker of unstable living conditions.

Our study was limited to one household visit, which prevented us from collecting data on the long-term outcome of the pediatric index cases enrolled. Due to the cross-sectional nature of the study, we were unable to follow the 14 new TB suspects to see whether they were eventually classified as TB cases. Finally, while the symptoms used to screen for possible household TB cases are appropriate for identification of adult TB suspects, this may not be a sufficient measure for identification of household pediatric TB suspects.

## 5. Conclusions

Contact tracing is a proven strategy for preventing the spread of tuberculosis. In our study of 50 pediatric index cases newly diagnosed with TB disease in Pune, India, we identified 14 additional TB suspects living in their homes. The tool, however, is gravely underutilized among our study population. Many of the children in the study had known TB cases in their households prior to their own diagnosis but had not been offered IPT, despite national guidelines calling for such treatment. The practice of investigating only household contacts may be inadequate in urban slum populations. Most of the TB cases we investigated seem to have come from outside the home. Future large-scale studies should address the efficacy and feasibility of including community contacts in TB contact investigation, especially in countries with high population density and high TB burden, like India.

## Figures and Tables

**Table 1:** Sociodemographic and clinical characteristics of pediatric index cases.

Characteristic	Total enrollees (*n* = 50)	Cases with known household TB contacts (*n* = 15)	Cases without known household TB contacts (*n* = 35)	*P* value

*Personal information *				
Gender				0.41
Male	29 (58%)	10 (67%)	19 (54%)	
Female	21 (42%)	5 (33%)	16 (46%)	
Median age, months (IQR)	27 (18–38)	27 (18–45)	28 (18–48)	0.89
Daily contact with people outside home	36 (72%)	10 (67%)	26 (74%)	0.73
Neighbors	32 (64%)	10 (67%)	22 (63%)	
Attends school	12 (24%)	3 (20%)	9 (26%)	
Extended family	9 (18%)	1 (0.1%)	8 (23%)	
Other	3 (6%)	0 (0%)	3 (0.09%)	
HIV status				0.54
Positive	9 (18%)	4 (27%)	5 (14%)	0.29
Negative	36 (72%)	10 (67%)	26 (74%)	
Unknown	5 (10%)	1 (0.1%)	4 (11%)	
Median time between TB diagnosis and start of TB treatment, days (IQR)	3 (0–15)	17.5 (0–26)	2 (0–9)	0.03
*Household information *				
Location of house				0.29
Urban/periurban	46 (92%)	14 (93%)	32 (91%)	
Rural	2 (4%)	1 (0.07%)	1 (0.03%)	
Other	2 (4%)	0 (0%)	2 (0.06%)	
Family type				0.85
Joint	29 (58%)	9 (60%)	20 (57%)	
Nuclear	21 (42%)	6 (40%)	15 (43%)	
Median adults in the home (IQR)	3 (2–5)	3(2–6)	3 (2–5)	0.74
Median children in the home (IQR)^a^	2 (1–3)	2 (1-2)	2 (2-3)	0.05
House with ≤2 rooms	42 (84%)	12 (80%)	30 (86%)	0.68
House with no windows	32 (64%)	9 (60%)	23 (66%)	0.70
House with 1 person employed for pay	24 (48%)	7 (47%)	17 (49%)	0.90
Monthly household income^b^				0.82
$0–63 USD	17 (34%)	6 (40%)	11 (31%)	
$63–111 USD	19 (38%)	4 (27%)	15 (43%)	
>$111 USD	12 (24%)	4 (27%)	8 (23%)	
Refused to Answer	2 (4%)	1 (0.06%)	1 (0.03%)	
Primary caretaker education level ≤4th grade	19 (38%)	4 (27%)	15 (43%)	0.35
Moderate to severe food insecurity	12 (24%)	7 (46%)	5 (14%)	0.01

INR: Indian Rupees; IQR indicates interquartile range.

^a^The index case.

^b^Based on conversion of 1 USD = 48.5 INR (average for 2010-2011) [4].

**Table 2:** Characteristics of pediatric index cases by HIV status.

Characteristics	Total (*n* = 50)	HIV-positive (*n* = 9)	HIV-negative^a^ (*n* = 36)	HIV status unknown (*n* = 5)	*P* value
*Tuberculosis risk factors *					
Household contact with confirmed TB	15 (30%)	4 (44%)	10 (27%)	1 (20%)	0.42
Household contact with TB symptoms	10 (20%)	3 (33%)	6 (16%)	1 (20%)	0.35
Received IPT in the past	1 (2%)	1 (11%)	0 (0%)	0 (0%)	0.20
Received BCG vaccine	48 (96%)	7 (77%)	36 (100%)	5 (100%)	0.03
Type of cook stove					0.12
Gas	12 (24%)	2 (22%)	6 (16%)	4 (80%)	
Kerosene	27 (54%)	3 (33%)	24 (66%)	0 (0%)	
Biomass	11 (22%)	4 (44%)	6 (16%)	1 (20%)	
Smoker in the home	7 (14%)	0 (0%)	7 (19%)	0 (0%)	0.31
*Tuberculosis history *					
Symptoms present at diagnosis^b^	46 (92%)	9 (100%)	33 (91%)	4 (80%)	0.37
Positive diagnostic test for TB	50 (100%)	9 (100%)	36 (100%)	5 (100%)	0.83
Mantoux	26 (52%)	4 (44%)	20 (56%)	2 (40%)	
Sputum	4 (8%)	0 (0%)	2 (0.06%)	2 (40%)	
Gastric aspirate	1 (2%)	0 (0%)	1 (0.03%)	0 (0%)	
Chest X-ray	27 (54%)	6 (67%)	17 (47%)	4 (80%)	
Ultrasound	22 (44%)	6 (67%)	15 (42%)	1 (20%)	
Type of TB diagnosed^c^					0.40
**Confirmed TB cases**	0 (0%)	0 (0%)	0 (0%)	0 (0%)	
**Probable TB cases **(*n* = 5)					
Pulmonary TB	3 (60%)	0 (0%)	2 (67%)	1 (50%)	
Extrapulmonary TB	0 (0%)	0 (0%)	0 (0%)	0 (0%)	
More than 1 type of TB	2 (40%)	0 (0%)	1 (33%)	1 (50%)	
**Possible TB cases **(*n* = 45)					
Pulmonary TB	16 (35%)	2 (25%)	11 (34%)	3 (60%)	
Extrapulmonary TB	17 (37%)	2 (25%)	14 (43%)	1 (20%)	
More than 1 type of TB	10 (22%)	3 (37%)	6 (18%)	1 (20%)	
Type of TB not recorded	2 (4%)	1 (12%)	1 (3%)	0 (0%)	
Treated with Category 1^d^ medications	49 (98%)	9 (100%)	35 (97%)	5 (100%)	0.61
Completed treatment	2 (4%)	0 (0%)	2 (5%)	0 (0%)	0.63
Difficulty in obtaining medications	7 (14%)	1 (11%)	6 (16%)	0 (0%)	0.68

BCG: bacillus Calmette-Guérin vaccine, HIV: human immunodeficiency virus, IPT: isoniazid preventive therapy, TB: tuberculosis.

^a^Six of the 50 children had unknown HIV status.

^b^TB symptoms of cough, fever, weight loss, or night sweats.

^c^Two of the index cases did not specify what type of TB (1 HIV-positive case and 1 HIV-negative case).

^d^Category I is isoniazid, rifampin, ethambutol, and pyrazinamide for two months followed by isoniazid and rifampin for four months.

Category II is isoniazid, rifampin, ethambutol, pyrazinamide, and streptomycin for two months followed by isoniazid, rifampin, ethambutol, and pyrazinamide for one month followed by isoniazid, rifampin, and ethambutol for five months.

Category III is isoniazid, rifampin, and pyrazinamide for three months followed by isoniazid and rifampin for four months.

**Table 3:** Characteristics of prevalent TB cases and new TB suspects in the household.

	Confirmed, known TB cases (*n* = 16^a^)	TB suspects (*n* = 14^b^)

*Relation to pediatric source case *		
Parental caregiver	11 (68%)^c^	3 (21%)
Sibling	2 (12%)	7 (50%)
Other family	3 (18%)	4 (28%)
*Children *	2 (13%)	8 (57%)
Median age, years (IQR)	6.5 (6.2–6.7)	4.0 (2.0–6.0)
HIV-positive	0 (0%)	0 (0%)
HIV status unknown	0 (0%)	5 (35%)
*Adults *	14 (87%)	6 (42%)
Median age (IQR)	30 (24–34)	30 (25–46)
HIV-positive	4 (25%)	2 (14%)
HIV status unknown	0 (0%)	2 (14%)
*Treatment history *		
Started TB treatment	15 (93%)	0 (0%)
Completed TB treatment	7 (43%)	0 (0%)
Given isoniazid preventive therapy	0 (0%)	0 (0%)

HIV: human immunodeficiency virus, IQR: interquartile range, TB: tuberculosis.

^a^One child had 2 known TB cases in the household.

^b^Two children each had 2 TB suspects in each of their homes.

^c^Six maternal and five paternal caregivers.
